# Multidisciplinary surgical management of Cowden syndrome: Report of a case

**DOI:** 10.4317/jced.52919

**Published:** 2016-10-01

**Authors:** Romeo Patini, Edoardo Staderini, Patrizia Gallenzi

**Affiliations:** 1 DDS, Department of Surgical sciences for head and neck diseases, School of dentistry, Catholic University of Sacred Heart, Dean: Prof. Massimo Cordaro, Largo A. Gemelli, 1 - 00168 Rome, Italy; 2 Student, Department of Surgical sciences for head and neck diseases, School of dentistry, Catholic University of Sacred Heart, Dean: Prof. Massimo Cordaro, Largo A. Gemelli, 1 - 00168 Rome, Italy

## Abstract

Cowden’s Syndrome (CS) is a rare congenital autosomal dominant disorder that affects around 1/200000 patients with an incomplete penetrance and variable expressivity, characterized by alterations in a tumor suppressor gene. A 14-year-old Caucasian male patient came to the attention of the authors complaining of palm nodules, gingival bleeding and painful pedunculated lesions on the lips and on the labial side of anterior sextants. After genetic investigation the final diagnosis of a Cowden Syndrome was made. The lesions were surgically removed under general anesthesia and no clinical signs of recurrence were found three months after surgical excision. Considering the severe symptoms of the syndrome and the strong tendency to malignant development of the associated lesions all clinicians should focus their efforts to the early diagnosis and, when possible, multidisciplinary treatment.

** Key words:**Early diagnosis, multiple hamartoma syndrome, oral papillomatosis, cancer predisposition, case report.

## Introduction

Cowden’s Syndrome (CS) is an autosomal dominant disorder that is part of over 200 “highly penetrant hereditary cancer syndromes” that affects around 1/200000 subjects (1). CS is an incomplete penetrance and variable expressivity congenital disease associated with genetic alterations in PTEN (Phosphatase and TENsin homolog), a tumor suppressor gene. Intragenic germline PTEN mutation is also recognized in Bannayan-Riley-Ruvalcaba Syndrome (BRRS), Proteus syndrome and Proteus-like syndrome. Considering their common genetic etiology, the aforementioned congenital disorders are grouped as PTEN hamartoma tumour syndrome (PHTS). Clinical features of CS are extensive proliferation of multiple hamartomas from all germinative lineages and a multi-organ increased predisposition to different tumors so that it is estimated that 39.1% of affected patients are diagnosed at least one malignant tumor (2). A relevant association with immunodeficiency has recently been described (3).

## Case Report

A 14-year-old child came to the attention of the Operative and Paediatric Dentistry Unit of the Department of Surgical Sciences for Head and Neck Diseases – teaching hospital “Agostino Gemelli - University of Sacred Heart” of Rome complaining of gingival bleeding and recently painful pedunculated lesions on the labial side of anterior sextants.

Medical history of the patient revealed that he was diagnosed a medulloblastoma, treated with surgery and chemotherapy, at age 2. In August 2004 a cancer recurrence was observed and treated with radiotherapy followed by chemotherapy. With the onset of abdominal discomfort and bloating multiple upper and lower gastrointestinal endoscopies were performed: the exams showed multiple non-dysplastic colon polyps and small gastric and duodenal growths. In order to prevent further disease manifestations, TC-scan was performed and it revealed nodular lesions involving lungs and liver. The presence of cutaneous facial papules and oral mucosal papillomatosis, present in 99 to 100% of cases, represent a notable features of CS semeiological pattern; so, basing on criterium 1b of International Cowden Syndrome Consortium diagnostic work-up, the clinicians put the final diagnosis of Cowden Syndrome. For further confirmation a genetic test was prescribed to give additional information about the patient’s disease: through DNA sequencing and denaturing high performance liquid chromatography (dHPLC) a PTEN germline mutation c. 697 C<T (R233X) was identified.

**Figure 1:**
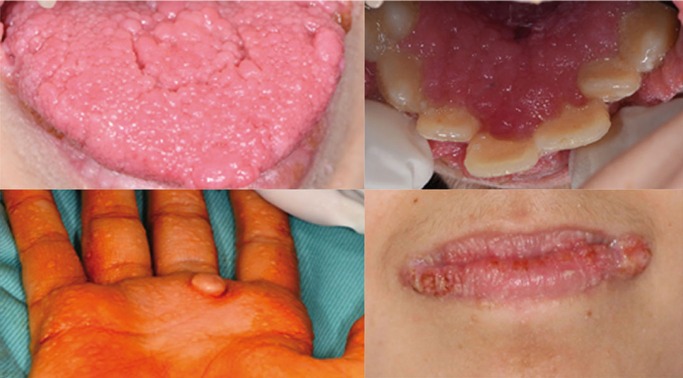
Photographs showing grooves and enlargements of the tongue surface, oral multiple coalescent papillomatous lesions covering cervical area of teeth, prominent lesions affecting lip angles, hand palm lesion and palmar keratosis.

Dental examination revealed multiple widespread carious lesions (DMFT index: 2+1+8= 11), dental malposition and premature teeth loss of the first right lower premolar. Intraoral mucosal examination disclosed a tongue morphology altered by grooves and multiple, confluent, asymptomatic smooth surfaced, sessile or pedunculated non-tender papules of variable size (ranging from 1 to 3 mm in diameter), also localized on lips, angles of the mouth, retrocommissural areas, buccal mucosa and attached gingiva; hard palate and dorsum of tongue, moreover, showed a firm consistency surface of “cobblestone” appearance. Periodontal screening disclosed poor oral hygiene (Oral Hygiene Index: 5.5) associated with plaque and bleeding scores above cut off levels, hyperplasic and hyperemic gingival margin with generalized pseudopockets (especially on palatal side of the anterior sextant) and foetor ex ore. There was no clinical evidence of potentially malignant lesions in the oral district (Fig. 1).

**Figure 2:**
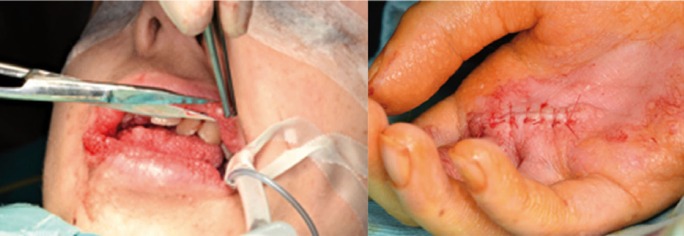
Images showing surgical excision of multiple papules affecting the patient’s upper and lower lips and the hand palm.

**Figure 3:**
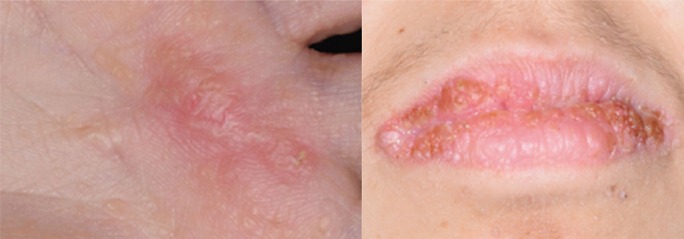
3-months clinical follow-up of hand palm excisional biopsy and lip surgical wounds.

After preliminary anesthetic assessment was made and confirmation of informed consent was obtained the general anesthesia was induced with Propofol (4 mg/kg) and maintained with Desflurane and, as an adjunct to general anesthesia, Rocuronium bromide injection (0,25 mg/kg) was performed in order to provide skeletal muscle relaxation during surgery and to facilitate tracheal intubation. Surgical area was cleaned with Povidone iodine 10% and palm nodule and lip lesions were removed through traditional scalpel excision (Fig. 2). Since patients affected by CS are considered at high cancer predisposition, although no evidence of malignancy was found, clinicians decided to perform an excisional biopsy of the hand palm lesion in order to histologically corroborate the clinical evidence. Surgical wounds were closed with a few single interrupted sutures using 5-0 fast resorbable Polyglactin 910. The specimens were placed in 10% neutral buffered formalin (more than 20 times the volume of the sample to avoid improper fixation or autolysis). All the surgical wounds healed with primary closure; no recurrence was seen at the 3 months clinical follow-up (Fig. 3).

## Discussion

Cowden’s syndrome, or multiple hamartoma tumour syndrome, was first described in 1940 by Costello in a 27-years old Mexican female who later died at the age of 47 for breast cancer (4). Lloyd and Dennis defined and named this syndrome after their patient Rachel Cowden, a 20-years old female with adenoid facies, high-arched palate, hypoplasia of soft palate and uvula, papillomatosis of the lips and oropharinx, scroted tongue, thyroid lesions, hypertrophy and fibrocystic disease of the breast, scoliosis, lesions of bone and liver (5).

The origin of CS has been supposed being T-cell, NK-cell or viral related (2). In 1978 the autosomal dominant transmission of this syndrome was confirmed. In 1996 Nelen *et al.* firstly identified the gene associated with Cowden syndrome on chromosome 10q22-23 (6). One year later Li *et al.* established the exact location of the gene on chromosome 10q23.3 giving it the name of PTEN (7).

In the clinical management of CS the oral specialist may play an important role in the early recognition of the disease, surgical care and lifelong follow-up. In fact dentists must be aware that CS is a progressive pathological syndrome involving orofacial and mucocutaneous manifestations in an early stage and must refer the patient for a complete investigation in suspected cases (8).

Surgical excision of mucocutaneous lesions can be performed as a treatment option for aesthetic reasons or functional problems, such as pain or removable prosthesis displacement. Furthermore, biopsied oral lesions can help the clinician to reject the risk of underlying malignancies and to monitorize the possible evolution of suspected hamartomas. After diagnosis clinicians should remember to the patient the importance of frequent multisystem surveillance and prompt intervention of symptomatic lesions that, if untreated, can increase the risk of malignancy.

A recent association of CS with impaired immunological function recommends increased clinical effort in screening and prevention, owing to high susceptibility to infections (3).

## Conclusions

Due to multiple organ system involvement of CS, matching different signs and symptoms should be the key to make the diagnostic process easier and faster. In light of the severe semeiological features of the syndrome and the risks related to anesthetic procedures (9), it is worthwhile to develop a proper skills integration between health care professionals in preventive and treatment measures in order to plan a comprehensive treatment. Since owing to continued surgical treatments and regular cancer screenings the affected patients’ compliance substantially decreases becoming low enough to induce desperately to refuse essential interventions, the general anesthesia approach, as demonstrated in this case report, should be consider a valid therapeutic option because it allows to treat simultaneously different anatomical district with high accuracy. This multidisciplinary one-step approach is expected to give better results than separated procedures, especially for children and phobic patients.
